# A PLC-based pomegranate sprout removal device design

**DOI:** 10.1038/s41598-024-55068-8

**Published:** 2024-03-01

**Authors:** Liqi Qiu, Jingbin Sun, Xieliang Zhang, Qun Sun, Ying Zhao

**Affiliations:** School of Liaocheng University, Mechanical and Automotive Engineering, Liaocheng, 252000 China

**Keywords:** Engineering, Mechanical engineering

## Abstract

Aiming at the current low degree of mechanization of pomegranate sprouting tiller pruning in China, all relying on manual pruning, this paper designs a PLC-based pomegranate sprouting tiller removal machine. This machine adopts the identification method of wireless map transmission, the sprouting tiller removal method of multi-cylinder cooperative operation, and the MCGS configuration to realize the interaction between the user and the system, which realizes the displacement and angle compensation of the end-effector under complex conditions to realize the all-around accurate removal of the pomegranate sprouting tiller. The performance test and finite element analysis showed that the device could remove up to 74.62% of sprouting tillers, and the damage rate was as low as 18%. This meets the requirements of pomegranate plantations for the removal of emergent tillers.

## Introduction

Pomegranate is known for its nutritional, medicinal, and ornamental importance^1^. The fruit is popular because of the organoleptic characteristics of its arils as well as its nutritional and therapeutic value, which are useful in the treatment of cancer, indigestion, leprosy, and various chronic diseases^2–4^. The plant has a high adaptive capacity; as a crop, pomegranate is widely cultivated in tropical and subtropical areas^5^. If pomegranate trees are not pruned and are allowed to grow naturally, they often sprout from the rhizosphere. Sprouted tillers do not bear fruit, but only consume nutrients, slow down the growth of pomegranate trees, and have no effect on high-yield tree structure; this type of tiller will also cause disorder of branches in the crown and poor illumination in the inner chamber of the tree, which may not only cause the fruit tree to grow leaves without flowering but also make the fruiting part move outward, and the branches will be weak and die, which will eventually lead to premature aging of the fruit tree^6^. The reasonable pruning of pomegranate trees can effectively control the growth of sprouting stumps. Controlling the growth of corms can make the branches of pomegranate trees sparse and dense, improve the flowering rate, fruiting rate, and yield of pomegranate trees, and extend their fruiting period of pomegranate trees^7^. There are too many tillers to be pruned for pomegranate planted on a large scale; therefore, it is difficult to prune the tillers. Currently, manual pruning is the most commonly used^8,9^. Manual tillering is inefficient, costly and dangerous^10,11^. At present, there is no specific device for removing the sprouting of pomegranate trees. One of the main methods is manual tillering, which uses a knife, branch scissors, or electric scissors to prune sprouting from the trunk. This method can completely remove the sprouting without causing damage to the trunk of the tree, but it is time-consuming and difficult to prune pomegranate trees at high places^10^. In addition, the branches of pomegranate are complex in structure, and the distribution angles and orientations of sprouting tillers are uneven. Branch scissors and electric scissors cannot completely remove sprouting tillers with complex growth, and they are not suitable for large-scale pomegranate planting. Considering the shortcomings of poor wear resistance, a pneumatic trimmer must be developed, designed, and optimized urgently^12^. To reduce the manual labor force and realize the mechanization of pomegranate sprout picking. Therefore, this machine has been developed to realize the shaping and pruning of pomegranate trees all year round and has the advantages of replacing a lot of manual work and long-term use, which will effectively reduce the cost of large-scale pomegranate planting, thus increasing farmers' income.

Pomegranate sprouting and picking belong to the field of pruning, which mostly uses backpack type pruning machines. Ma Wenbin et al.^13^ developed a backpack type branch pruning machine represented by a multi-angle pruning saw for landscaping and greening. This type of backpack pruning machine mostly uses gasoline engines as the power source and has low costs and good working effects; however, it still requires manual carrying, with high work noise, strong vibration, and difficulty in the market, Meng Chao et al.^14^, designed a new type of lifting auxiliary lifting platform represented by a multi degree of freedom high branch pruning manipulator, which has a high degree of automation, good work efficiency and effectiveness, and is suitable for planting large-scale orchards. However, because of its large size and high price, it is not suitable for many small farmers who plant scattered crops; Wang Jichen^15^ and others studied and designed tree-climbing robots, Yang Xi^16^ and others, and developed and designed tree-pruning robots. Meng et al.^17^ developed a raspberry-pruning robot that collects sprout information through a camera, thereby directing the tool to move to the sprout for cutting. However, the end effector of this type of machine cannot move accurately, and the growth structure of pomegranate trees is complex, with branches intertwined and unable to locate the cutting point accurately. In the field of fruit tree pruning in hilly areas. The pruning device for peach trees in hilly areas developed by Ruihua et al.^18^, is simple to operate and has good cutting effects. However, this type of device uses a circular saw blade pruning method, which can damage the trunks of pomegranate and other trees when pruning.

Many universities and enterprises in China have conducted research on the selection of pomegranate sprouts. Wang Shuyuan^19^ and others from Nanjing Forestry University developed a handheld pomegranate tree root tiller trimmer. This device can control the size of the opening of the trimmed cover according to pomegranate trees with different diameters. The power motor drives the trimming blade to rotate at high speed. Trimming the root tillers of pomegranate trees can effectively improve working efficiency and reduce working intensity. However, the sprouting effect of branches on the main stems of pomegranate trees is limited and cannot be effectively removed. Yaya et al. from Shihezi University studied a pruner that can be applied to pomegranate sprouts. The cutting range is large, the adaptability is good, and the operation is simple; however, the pose adjustment speed is slow, and the cutting efficiency is low.

To effectively alleviate the manual duplication of labor, reduce the labor burden, and improve the efficiency of cutting sprouts, this study designed a pomegranate sprout cutting machine with a simple structure, convenient operation, and powerful functions. Based on SOLIDWORKS software, the machine carries out three-dimensional digital design and the overall assembly of key components. It adopts PLC FX3U-32MT to control stepping motors, cylinders, and other related execution parts to realize the removal of sprouts and the removal of interfering branches and leaves. The MCGS configuration software was used to control the interface to control the actual operation of related structures. The cutting position was determined in real time to cut sprouts under different growth conditions. After experimental determination, the removal rate of sprouts by this equipment reached 74.62%, and the breakage rate reached 18%, which meets the requirements of pomegranate sprout removal.

## Methods

### Overall structure and workflow

**Figure 1:**
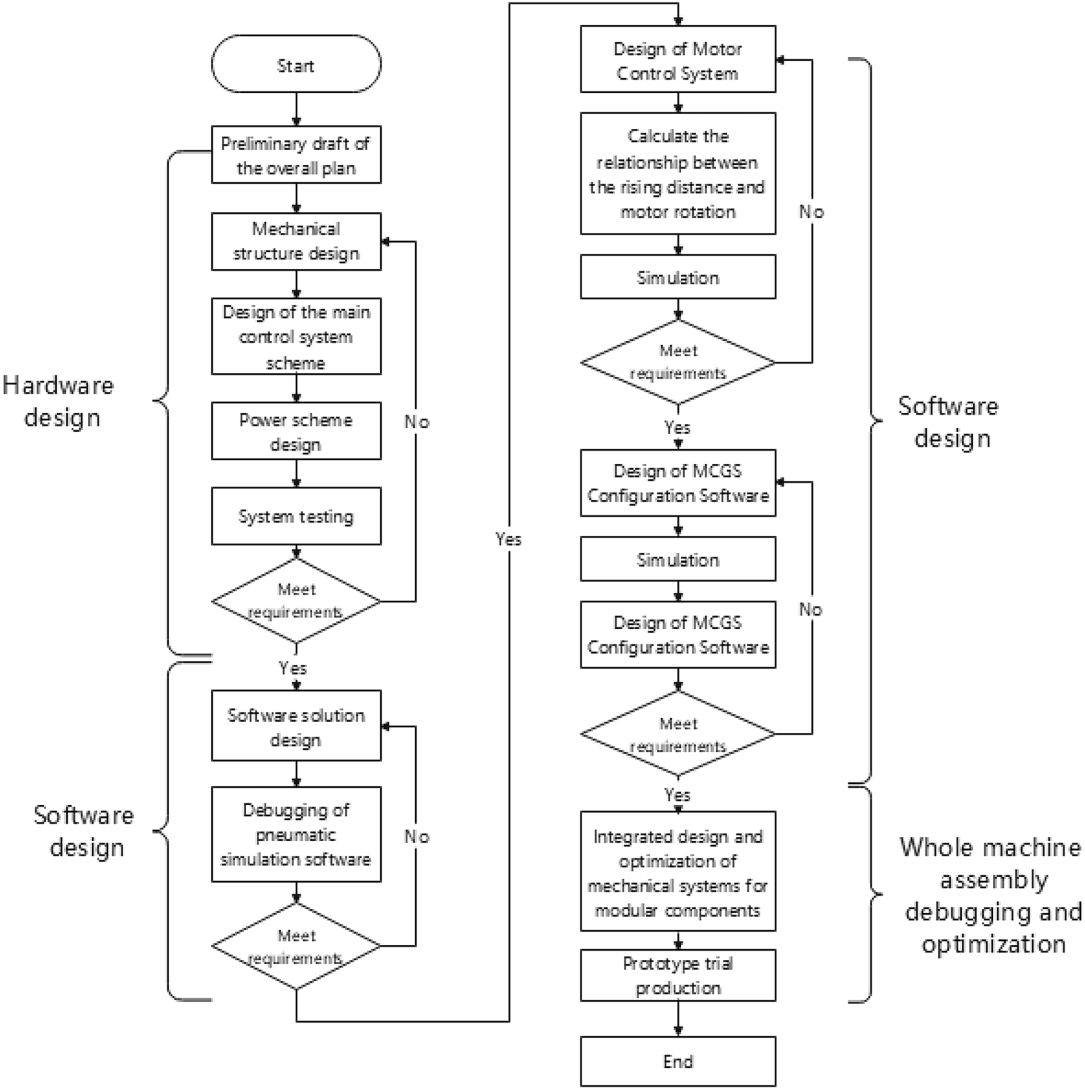
Technology roadmap of pomegranate sprout cutting machine.

This study aims to design a pomegranate sprout cutting device based on PLC. First, through on-site investigation of the growth height of pomegranate trees, diameter of pomegranate sprouts, and height of most sprouts, combined with existing sprout cutting equipment on the market, SolidWorks 2020 was used to establish a model of the relevant structure of pomegranate tree sprout cutting equipment. Then, FluidSIM-P 3.6 pneumatic simulation software was used to conduct simulation experiments on the pneumatic part of the equipment and the electrical control part that controls the reversal of the pneumatic part; second, the electrical control part and stepper motor control part were connected to the corresponding parts of the FX3U-32MT PLC, control was simulated through GX works2 programming software, and a monitoring interface was created through MCGS configuration software 6.2. Transfer the produced monitoring interface to the FX3U-32MT PLC and then enter the operating environment for the simulation control. Finally, debugging and operation of the overall key parts will be carried out, and the first-generation prototype will be manufactured and tested after the entire machine is correct. The technical research route used in this study is shown in Fig. 1.

**Figure 2:**
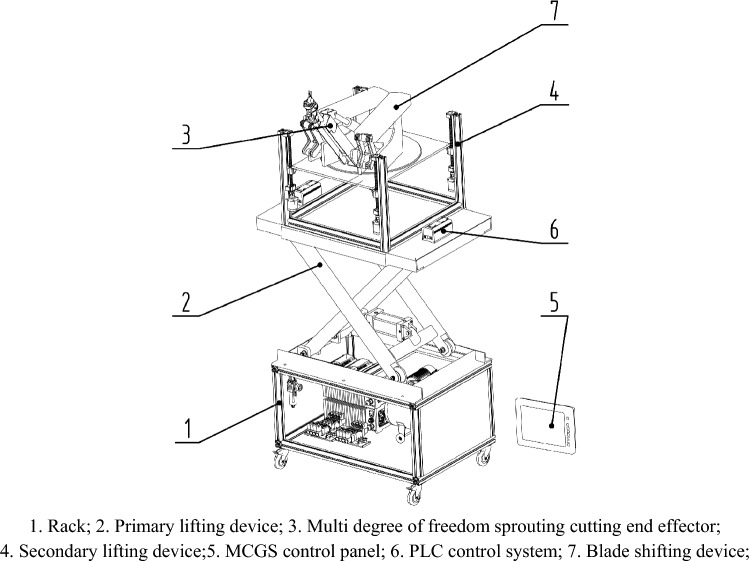
Overall three-dimensional structure diagram.

A PLC-based pomegranate sprout cutting machine was designed based on relevant data of pomegranate trees and sprouts. The machine is mainly composed of a primary lifting device, secondary lifting device, multi degree of freedom sprout cutting device, leaf-shifting device, pneumatic control system, and MCGS control system. The overall three-dimensional mechanism is illustrated in Fig. 2. This device can perform image recognition based on equipped wireless image transmission equipment to determine the growth position of sprouts on pomegranate trees in complex states. Then, through the manual operation of the MCGS control panel, the first-level lifting platform can be roughly adjusted higher, and the second-level lifting platform can be precisely adjusted higher. Furthermore, a multi degree of freedom cutting mechanism can be controlled to complete the cutting of sprouts in complex states.

The working process of the device is divided into four stages:

First, the lowest machine was pushed to the bottom of the pomegranate tree by humans.

1. The camera identifies the location of pomegranate sprouts and determines the sprouts and their positions that need to be removed;
2. The camera provides real-time feedback of sprout images to the screen connected to the MCGS screen and adjusts the primary and secondary heights by manipulating the MCGS buttons to ensure that the height is within the appropriate cutting range.
3. By controlling the MCGS control panel, the multi degree of freedom sprout cutting device was controlled to achieve the cutting of all sprouts that needed to be cut within this range.
4. After the cutting was completed, the heights of the first and second levels were adjusted according to the actual cutting needs of the pomegranate tree sprouts. Repeat operation 3 and perform secondary cutting or multiple cutting.

Working principle: The first-level lifting platform uses a single cylinder for rough control of lifting position. The secondary lifting platform was controlled using four stepper motors to accurately reach the desired position. Mutual cooperation can achieve sprout removal at different height ranges.

### Workflow analysis and main technical parameters

**Figure 3:**
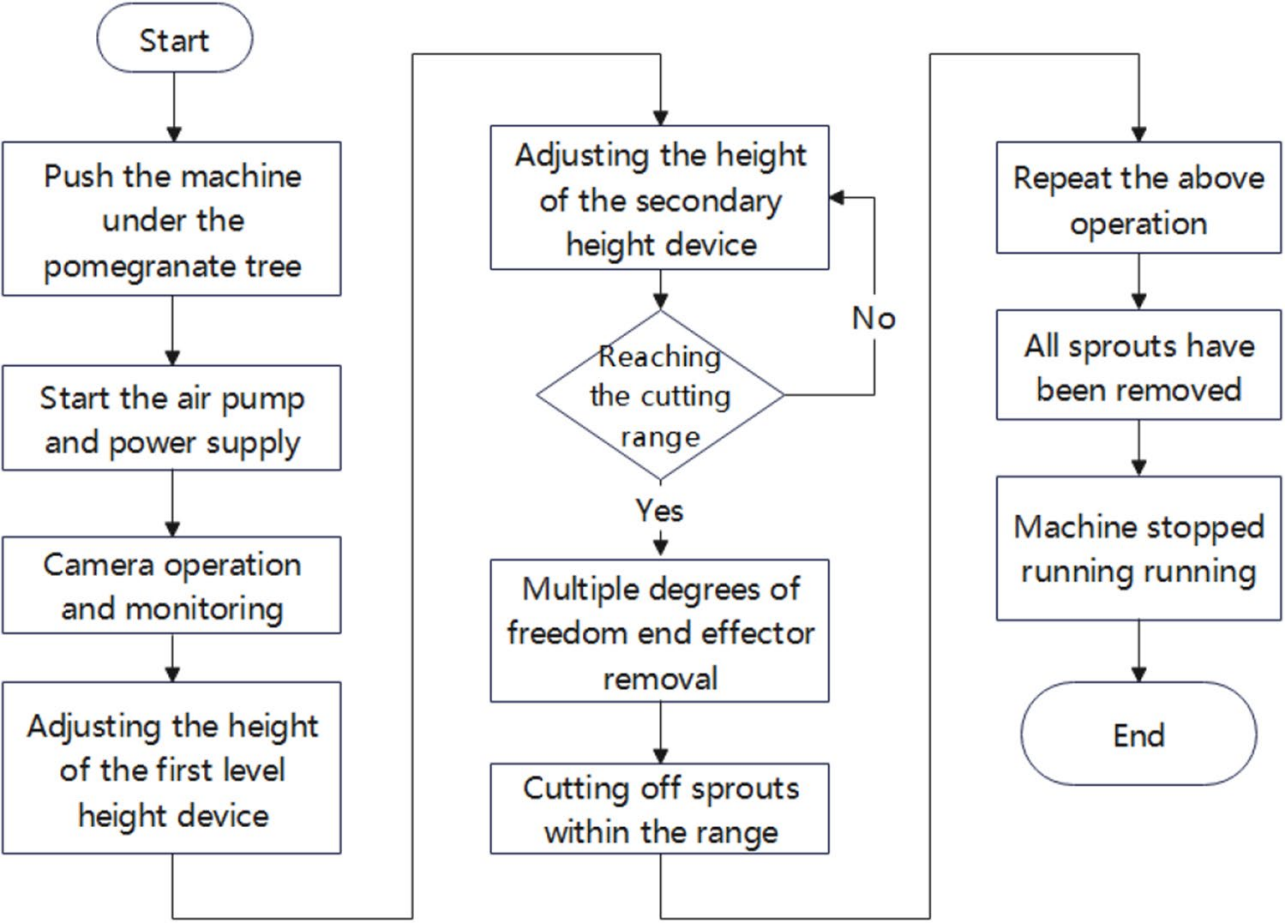
Working process of pomegranate sprout cutting machine.

The operator pushes the sprout cutting machine under the pomegranate tree, which must be cut off before leaving. The machine starts, and the real-time image transmission camera displays the position of the sprout in the pomegranate tree on a display screen connected to the manually controlled MCGS. Experienced staff operated the MCGS to determine the position of the sprout and then proceeded with sprout cutting. The specific removal process is as follows. The real-time image transmission function of the camera displays the position of the sprouts in real time, and when the nearest height position is manually determined, control and adjustment are performed. First, the first-level height lifting device is adjusted, the overall height is adjusted to the approximate position height, and then the second-level height adjustment is performed. The second-level height adjustment mechanism had a higher accuracy than the first-level adjustment mechanism. Through the cooperation of the first- and second-level height adjustment mechanisms, the height position of the end effector is adjusted to the appropriate range so that the end effector can cut all the required sprouts within the height range. After all sprouts that need to be cut off within the height range are cut off, the staff will perform the above operation again, raise the machine height to the next height range, and cut off the sprouts within the height range. When cutting: The end-effector structure adopts a multi cylinder collaborative multi degree of freedom end effector. The operator can control the operation panel on the MCGS to retract each cylinder, thereby achieving the goal of clearing all sprouts of pomegranate trees under various growth conditions. After the machine removed all sprouts of pomegranate that could be cut, the operator stopped the machine, stopped the air pump, and pushed it out to the appropriate position. The workflow is illustrated in Fig. 3.

The main technical indicators of the pomegranate sprout cutting machine are presented in Table 1.

**Table 1:** Main technical indicators.

Parameter	Numerical value
Device size (L * W * H)/(mm * mm * mm)	1600 × 800 × 800
Maximum device size (L * W * H)/(mm * mm * mm)	2500 × 1000 × 3000
Overall quality/(kg)	200
Secondary lifting device stepper motor power/(N m)	1.2
Motor power of rotary compensation device/(N m)	13
Working efficiency/(Tree/h)	Determined by the number of sprouts

## Design

### Design of primary lifting device

**Figure 4:**
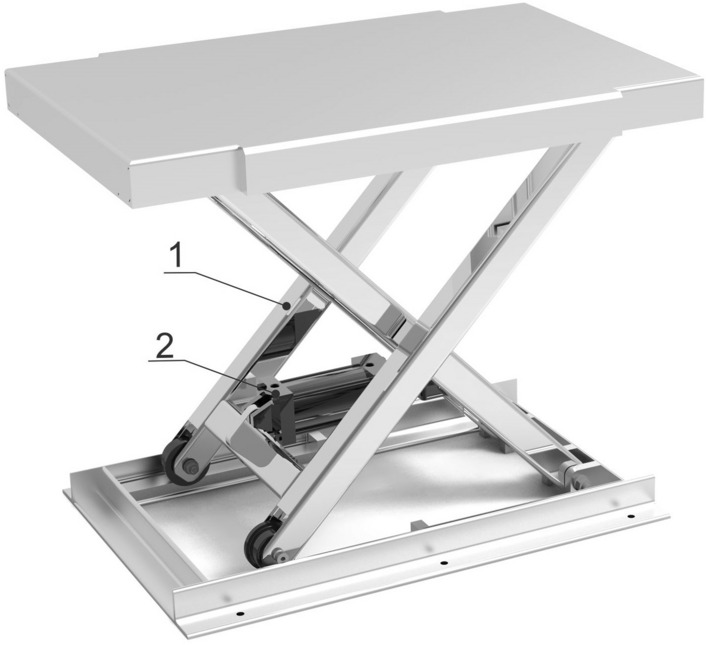
Structure diagram of the three-dimensional model of the first level lifting device.

The first-level lifting device consisted of two sets of frames with rollers and cylinders, which were connected to the middle of the lifting device through fixed hinge supports. As shown in Fig. 4, the SC80-500 cylinder was selected as the power source, powered by an air pump, to perform rough lifting within the range, achieving the first-level height adjustment of the pomegranate sprout cutting machine to most of the sprout growth range, and laying a certain foundation for the subsequent second-level precise lifting device to find suitable cutting positions.

According to relevant data on pomegranate trees, the distribution range of pomegranate sprouts is 2 m, that is, the distance difference between the lowest and highest sprouts of pomegranate is 2 m. Therefore, the lifting range of the cutting machine should be at least 2 m. Therefore, the design range of the first-level lifting device is approximately 1.2 m, and that of the second-level lifting device is approximately 0.8 m, which can meet the needs of most pomegranate trees for sprout removal.

**Figure 5:**
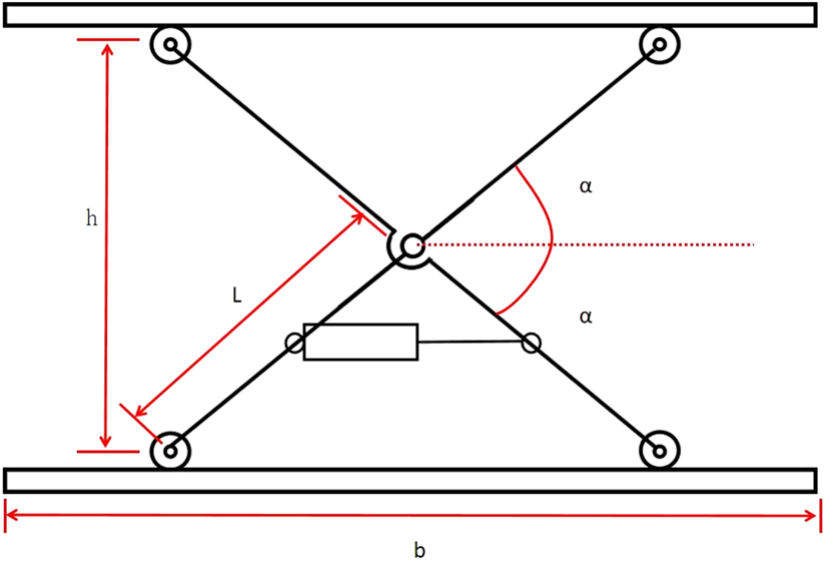
Schematic diagram of the structure and mechanism of the first level lifting device.

The range that the first level lifting device can provide for machine lifting is not less than 1.2 m. Based on this, combined with the relevant parameters of the cylinder and installation position, the bottom design size of the lifting device can be calculated, and then the overall size of the prototype can be designed to make its layout reasonable and structure reliable. A schematic of the mechanism can be obtained by drawing a three-dimensional model, as shown in Fig. 5.

To prevent the roller from detaching from the slide when the workbench is lowered to the lowest position, the length of the hinge plate 2 L should be smaller than the length b of the workbench, which is generally acceptable.

1 $$L = (0.45 - 0.48)b$$

From Fig. 5, it can be seen that h = 2Lsinα. It can be concluded that:

2 $$h_{\max} = 2L\sin \alpha _{\max}$$

3 $$h_{\min} = 2L\sin \alpha _{\min}$$

The effective vertical ∆h stroke of the lifting table is:

4 $$\Delta h = h_{\max} - h_{\min} = 2L\left( {\sin \alpha _{\max} - \sin \alpha _{\min} } \right)$$

Cylinder model SC80-450 was selected, with a length of 632 mm when contracted and a distance of 1082 mm when extended to the longest. The position of the cylinder was set in the middle of the lower bracket, as shown in the structural diagram above. Ignoring the length of the fixed hinge supports at both ends, it was assumed that the longest and shortest dimensions of the cylinder were the shortest and longest, respectively.

If the edge is d, then α can be used to represent d:

5 $$\cos \alpha _{\max} = \frac{{\left( \frac{L}{2} \right)^{2} + d_{\min}^{2} - \left( \frac{L}{2} \right)^{2} }}{{2 \cdot \left( \frac{L}{2} \right) \cdot d_{\min} }}$$

6 $$\cos \alpha _{\min} = \frac{{\left( \frac{L}{2} \right)^{2} + d_{_{\max}}^{2} - \left( \frac{L}{2} \right)^{2} }}{{2 \cdot \left( \frac{L}{2} \right) \cdot d_{\max} }}$$

Combining Eqs. (1)–(6), the maximum and shortest stretching distances of the known cylinder, as well as the distance of ∆h, should be taken into account. The value of ∆h should be ≥ 1.2 m, and a critical value of 1.2 m should be selected here.

The value of L can be calculated by incorporating the above formula into MATELAB:

*L* = 1135 mm.

According to Eq. (1), b = 2365–2552 mm. The connecting rod of the two fixed racks was set to a length of 2270 mm and the bottom surface was set to 2450 mm × 1000 mm.

### Rotating platform design

**Figure 6:**
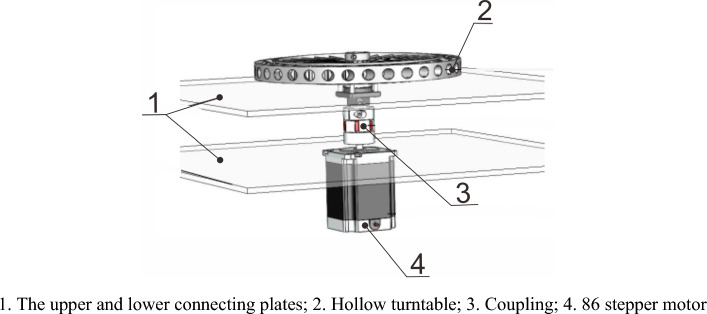
3D model of the mechanical structure of the rotating platform.

The design function of the rotating platform is to enable the multi degree of freedom end effector of the upper part to rotate around the z-axis, giving it a wider range of shear sprouts. Its structure is shown in Fig. 6. To ensure smooth operation of the rotating platform, a hollow turntable of the rotating platform was installed at the upper connecting plate, and the stepper motor was installed above the lower connecting plate. The upper and lower connecting plates were fixed to the slider of the screw module on the secondary lifting device with bolts to ensure the stability of the entire mechanism.

**Figure 7:**
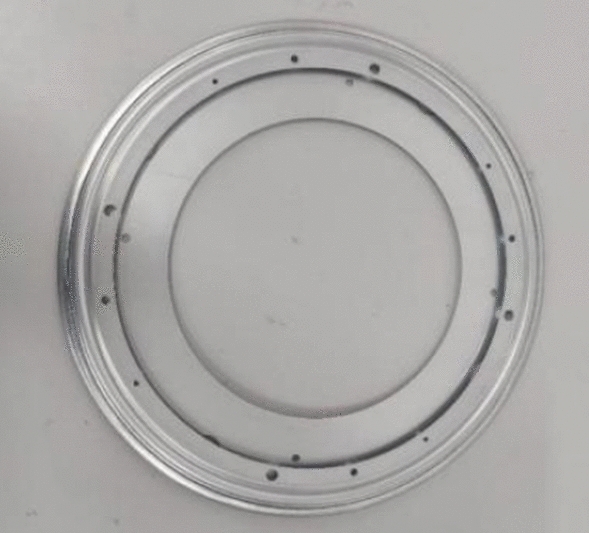
Physical image of hollow turntable.

To improve the practicality and reliability of the rotating platform, the rotating device adopts a 14 inch 360° galvanized circular hollow turntable with its top connected to a multi degree of freedom end effector. Then, the bottom of the end effector was welded to a connecting shaft and connected to the lower stepper motor through coupling. This allows the stepper motor to move the upper end effector in the same direction during the movement. Because the weight of the upper end effector is approximately 40 kg, considering safety and the additional force during rotation, a 14 inch hollow turntable was chosen, which can withstand a weight of up to 100 kg. The actual object diagram is shown in Fig. 7.

The rotational power of the rotating platform was provided only by a stepper motor connected to it. The selection of the fixed stepper motor is particularly crucial because the required accuracy of the stepper motor is not high, and its function here is only to rotate the rotating platform. When selecting the fixed stepper motor, only the step angle and torque were used for rough selection.

Firstly, the step angle α should satisfy:

7 $$\alpha \le \frac{\alpha _{\min} }{i}$$

In the formula, i is the transmission ratio;

α_min_ is the minimum angle required by the system for the components driven by the stepper motor.

Because the stepper motor is only directly connected to the welding shaft under the end effector through coupling, the transmission ratio i = 1 is fixed. Owing to the low accuracy requirement for the required rotation angle, the fixed angle only needs to be selected from the smaller step angles.

Second, for the stepper motor to operate normally (without losing step or stepping over), start normally, and meet the speed requirements, the starting torque T_q_ must meet

8 $$T_q \ge \frac{T_{m}}{{0.3 - 0.5}}$$

In the formula, T_m_ is the static load torque of the motor;

The load torque can be obtained from Eq. (9) as T_m_:

9 $$T_m = J \times \omega$$

where J is the Moment of inertia of the load;

To facilitate the calculation, all components of the upper end actuator can be converted into a solid cylinder to calculate its moment of inertia Jx. As mentioned earlier, the end effector mentioned above weighed approximately 40 kg, and 45 # steel was used as the manufacturing material, with a density of 7.85 g/cm^3^. It can be converted into a 45 # steel rod with a bottom radius of 300 mm and height of 22 mm. The Moment of inertia J can be obtained from Eq. (10):

10 $$J_x = \frac{1}{2}mr^{2}$$

Substituting the data into formulas (8)–(10). As the control here is an inching control, there is no angular acceleration. Press the touch screen to rotate the motor over a step angle corresponding to its angular acceleration. After taking the step angle of two-phase hybrid stepping motor and other relevant parameters, the starting torque T_q_ ≥ 10.8 Nm can be obtained. By searching for relevant tables, it can be seen that the selected stepper motor model is the 86BYG250H stepper motor with a holding torque of 12 Nm, which meets the usage requirements.

**Figure 8:**
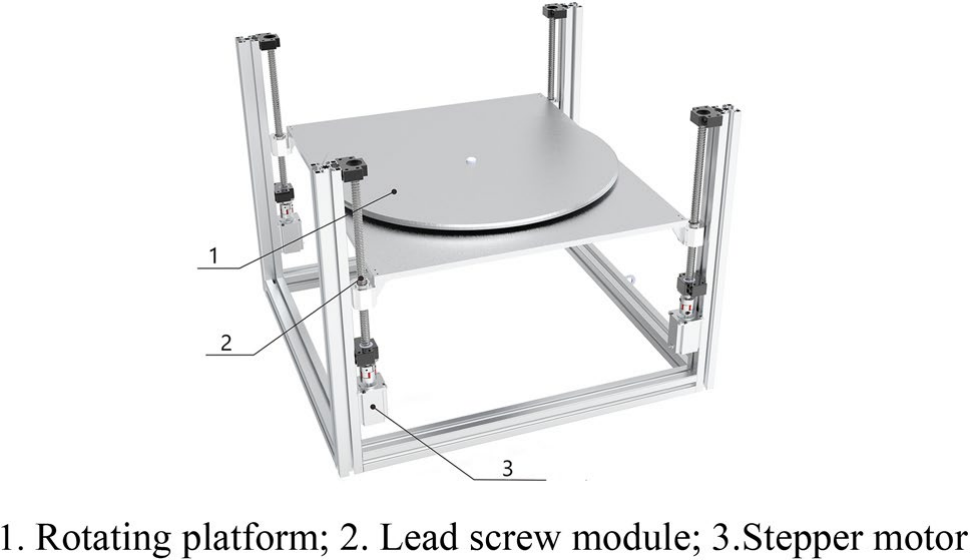
3D model of secondary lifting device.

The purpose of setting the secondary lifting device is to make slight height adjustments to the end effector when it reaches the approximate range where sprouts exist, to achieve a more precise height and achieve precise removal of sprouts. A 3D model image is shown in Fig. 8.

The mechanical structure of the secondary lifting device consisted of four screw modules and an aluminum profile frame. To set up this device, when the end effector runs to the approximate range where sprouts exist, slight height adjustments are made to achieve a more precise height, thereby completing the precise removal of sprouts. Therefore, the device only needs to ensure that it drives the end effector to rise and that the rising distance is within a small range each time.

**Figure 9:**
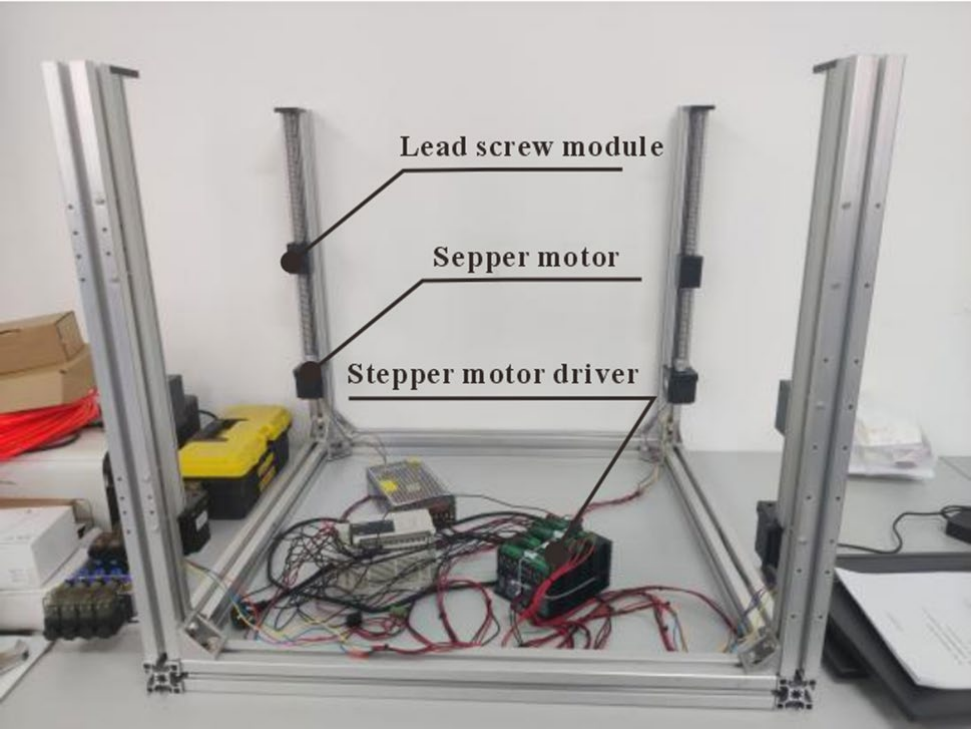
Physical image of the secondary lifting device.

As mentioned above, the total weight of the end effector, connecting plate, and stepper motor is approximately 50 kg. The SGX series single-track ball screw slide table was selected, and the model selection was 1605–600. The maximum vertical load of a single unit was 15 kg. Owing to the installation of four ball-screw modules in the device, the total weight that the fixed device can withstand is 60 kg ≥ 50 kg, which meets the requirements of the device design. In addition, the 1605 model represents a diameter of 16 mm for the screw and a pitch of 5 mm, which means that for every revolution of the stepper motor, the device increases by 5 mm, meeting the accuracy requirements for use. The specific physical framework construction is shown in Fig. 9, which shows the secondary lifting device connected to the stepper motor driver and the PLC.

### Design of pneumatic multi degree of freedom end effector device

**Figure 10:**
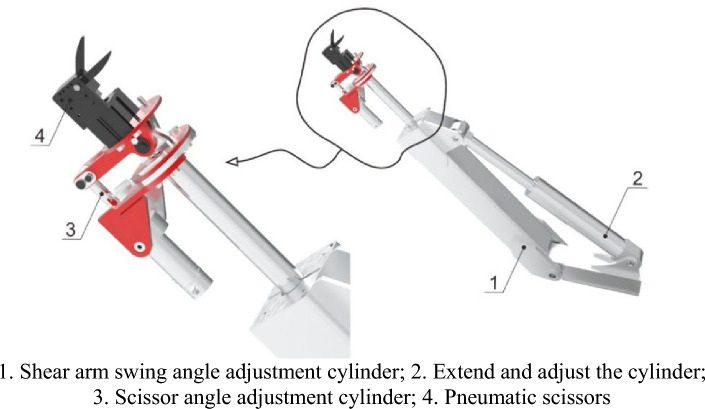
Partial enlarged view of pneumatic multi degree of freedom end effector and device.

The purpose of designing a pneumatic multi degree of freedom end effector is to achieve multi angle and all-round cutting of pomegranate sprouts. Its mechanical structure consists of three cylinders: the shear arm swing angle adjustment cylinder, extension adjustment cylinder, scissors angle adjustment cylinder, and pneumatic scissors set on the upper part of the extension adjustment cylinder, as well as the camera next to it. The three-dimensional mechanical structure model is shown in Fig. 10.

**Figure 11:**
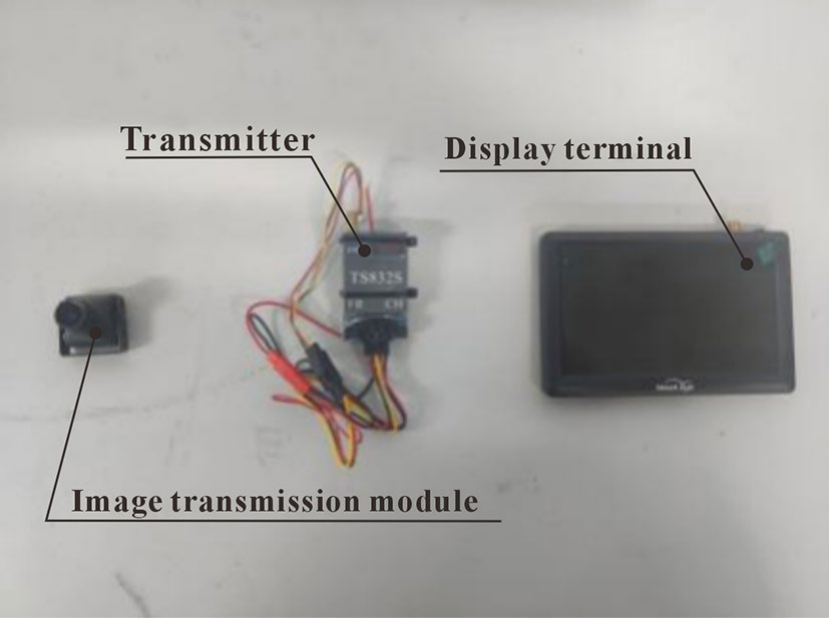
Physical image of camera and related modules.

The function of the camera in the end-effector device is real-time image transmission, which plays a role in real-time image feedback, determining the position of pomegranate sprouts, and facilitating machine cutting. It consists of a transmitter, an image transmission module, and a display terminal. The purchased physical image is shown in Fig. 11. The transmitter simulates and modulates the video signal and sends it wirelessly to the receiver, which demodulates and sends the signal to the display terminal. Select a 5.8G image transmission module, select the optimal eight channels for frequency modulation, with 20 frequency differences between adjacent channels, stable, efficient, and interference-free, with a small size, low power consumption, and high sensitivity. A camera is installed at the end effector. When the operator controls the end effector, the camera feeds back the environment inside the pomegranate branches and leaves, as well as the number and position of surrounding sprouts, to the screen. When there are sprouts in the pomegranate tree that need to be cut off on the screen, the operator can use the MCGS to control the cylinder and complete multi-angle sprout cutting.

Among the three cylinders of the pneumatic end effector, the most important consideration and selection design was the elongation adjustment cylinder. Because the cylinder is directly connected to the pneumatic scissors and related connectors, the force borne by the cylinder is the largest of the three cylinders, and its elongation distance directly determines the range of cutting off the sprouts of the end effector. Therefore, the selection and design of cylinders should be emphasized. According to the growth range of pomegranate sprouts, the position of the higher sprouts is about 2.5 m, and the height that can be lifted and lowered is determined to be 2 m by the first and second level lifting platforms mentioned above. The stroke of the designed elongation cylinder is 600 mm. When the shear arm swing angle adjustment cylinder is perpendicular to the machine plane, it can reach a maximum cutting height of 2.6 m, meeting the cutting needs of most pomegranate tree sprouts.

Using Festo's official online software for the simulation calculation, a stroke of 600 mm was selected, and the motion time was fixed at 30 s because of the device's jog control, which means the running distance per second was 20 mm. The air source pressure to 0.6Mpa, and the motion mass was set as pneumatic scissors and related connectors. It is roughly estimated to be 5 kg, set to the ideal state, and the friction force to be 0N. Using the DSBC-32-600-PSA-N3 cylinder for simulation, the relationship between displacement velocity and time, as well as acceleration and time, can be obtained, as shown in Table 2(a) and (b). After the simulation, it can be concluded that its motion time is 31.573 s, with an average velocity of 0.020 m/s, an end impact velocity of 0.021 m/s, and a maximum velocity of 0.150 m/s.

**Table 2:**
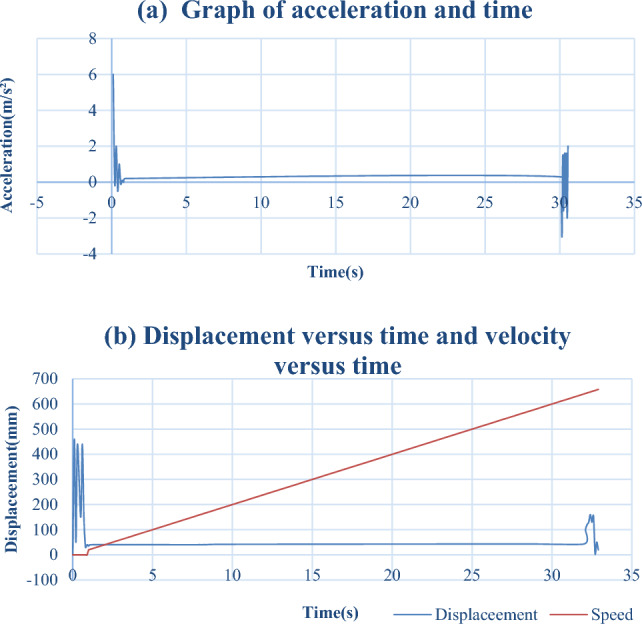
Graph of acceleration, time, and displacement versus time and velocity versus time.

**Figure 12:**
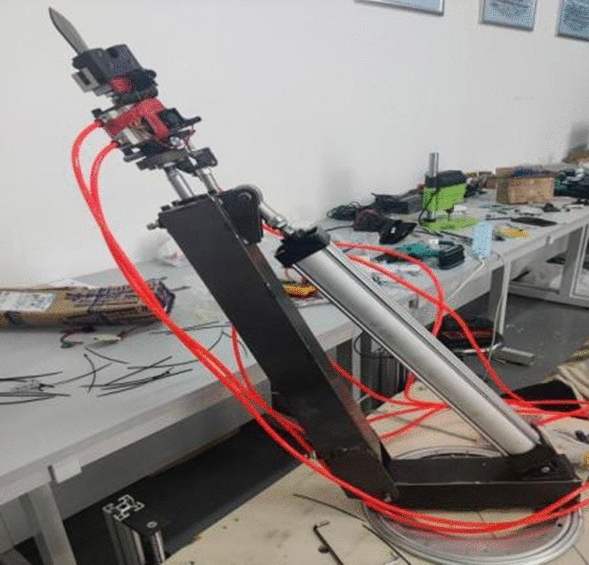
Physical diagram of multi degree of freedom pneumatic end effector.

From the above simulation calculations, it can be concluded that the point at which the cylinder is subjected to the maximum force occurs. As acceleration occurs at the moment of cylinder startup, it can be seen from Table 2(a) that the maximum value of acceleration, amax, is approximately 6 m/s^2^. As the load only drives pneumatic scissors, which is 5 kg, and the total weight of the cylinder, including the weight of the cylinder and the weight of the cylinder connecting parts, is about 20 kg, with a fixed Fmax of 150 N, According to the relevant manual of the cylinder, it can be seen that at 0.6 Mpa, the theoretical maximum force that the cylinder can withstand is 483N, which is greater than Fmax. Therefore, it is feasible to select this type of cylinder. A physical image of the pneumatic end-effector is shown in Fig. 12.

**Figure 13:**
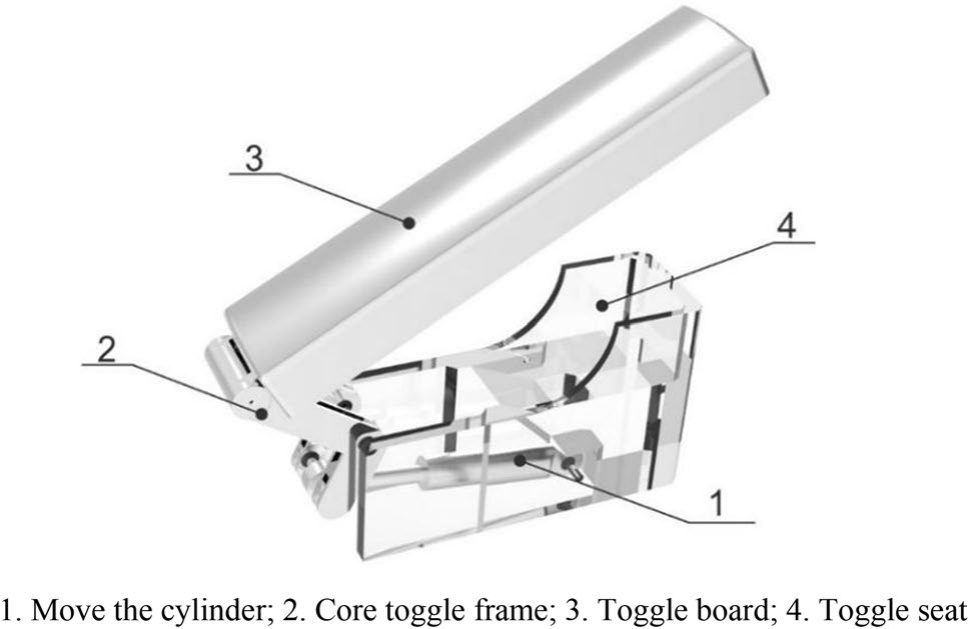
3D model of pomegranate sprouting and tillering assistant cutting device.

The function of the pomegranate sprout cutting auxiliary mechanism in the device is to move the branches blocked in front of the device so that the end actuator can cut the sprout more accurately. Its mechanical structure is composed of a toggle cylinder, core toggle frame, toggle plate, and toggle seat. By controlling the expansion and contraction of the cylinder, the toggle frame drives the toggle plate to make a circular motion to move the leaves and branches blocked in front. The three-dimensional model structure is shown in Fig. 13.

## Control system design

### Design of pneumatic circuit system

**Figure 14:**
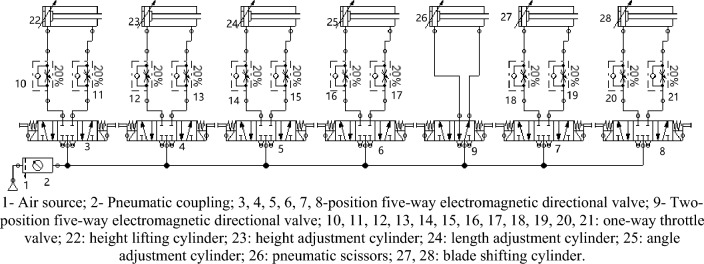
Pneumatic main circuit diagram.

The pneumatic circuit of pomegranate sprout cutting equipment uses high-pressure gas as the working medium for energy transfer. It controls the orientation of the end effector through three double-acting cylinders, achieving adjustment of the height, length, and angle of the end effector. The auxiliary blade-cutting device was controlled by two double-acting cylinders for blade shifting. The primary lifting function is achieved through a lifting cylinder, and pneumatic components, such as pneumatic scissors for cutting sprouts, are used as power devices to form a unified overall pneumatic circuit. Then, a programmable controller PLC FX3U-32MT was used to control the equipment. A total of six cylinders and one pneumatic scissor are required for the equipment. Through cylinder expansion control, the machine can cut off pomegranate sprouts in multiple directions, angles, and heights, and timely determine and move the position of sprouts and branches that need to be moved through the real-time image transmission system. The principle of the pneumatic main circuit of the equipment is illustrated in Fig. 14.

### Design and wiring of stepper motor system

The pomegranate sprout cutting equipment was based on a PLC FX3U-32MT control. In this PLC model, y0, y1, and y2 are the pulse output points in the PLC. Therefore, during wiring, one main 86 rotating platform stepper motor rotating platform and four secondary lifting device stepper motor lifting tables need to be connected to the pulse output points y0, y1, and y2, and y3, y4, and y5 are connected to the stepper motor direction port.

The pulse output point of the y0 output on the PLC FX3U-32MT is connected to the stepper motor pulse PUL on the stepper motor driver. Because COM1 on the PLC is connected to the 0v of V switching power supply, PUL + on the stepper motor driver is connected to 24 V, and the y3 output on the PLC is connected to the direction DIR—on the stepper motor driver. The DIR + on the stepper motor driver was connected to PUL + in parallel for 24 V. GND and V + are the working power supplies for the stepper motor drivers, and 24 V and 0 V are connected to the terminals of GND and V + , respectively.

The stepper motor driver has the advantages of increasing the output torque of the motor, a high starting frequency, strong driving reliability, low cost, and wide use. The stepper motor dial switches SW1, SW2, and SW3 output current settings, with peaks representing the full current and RMS representing the half current. Generally, the output current is in the form of half-current. SW4 control selects half of the current or full current output current. The SW4 dial is set to off to half the current output, the SW4 dial is set to on to full current output, and the SW5 SW6, SW7, and SW8 pulse output settings.

The 57 stepper motors selected for this device were 1.5A, with a step angle of 1.8°. The stepper motor sends every pulse to the stepper motor driver to rotate by 1.8 degrees. Therefore, in an ideal state, the stepper motor must send 200 (360/1.8) pulses for one revolution. However, in reality, the stepper motor output loses its steps. Therefore, to compensate for this problem, the stepper motor divides the step angle into M equal parts, One subdivision of 1.8/1 = 1.8 degrees sends 200 (360/1.8) pulse stepper motors for one revolution, two subdivision of 1.8/2 = 0.9 (360/0.9) degrees sends 400 (360/0.9) pulse stepper motors for one revolution, four subdivision of 1.8/4 = 0.45 degrees sends 800 (360/0.45) pulse stepper motors for one revolution, and so on. When debugging the equipment, we chose to send every 5000 pulse stepper motors for one revolution to improve the rotation accuracy of the stepper motor.

### Design of PLC control system for the entire machine

The system adopts PLC FX3U-32MT for cutting control of pomegranate sprouts, which requires the control of five stepper motors, four double-acting cylinders, and one pneumatic scissor.

According to the different input and output states, four two-stage lifting device 57 stepper motors that adopt collaborative control are selected for the equipment. Therefore, two of them are connected to the pulse output port of the same programmable controller, and the main 86 stepper motor are connected to the pulse port of the programmable controller. A PLC with three pulse output ports was required. Controlling the output status of the cylinder requires an electromagnetic directional valve as a control element, which requires a coil-type PLC. It is necessary to select the control hardware with a coil output port, and the input ports are all inputted using the MCGS touch screen. Therefore, two PLCs (FX3U-32MT) were selected.

**Figure 15:**
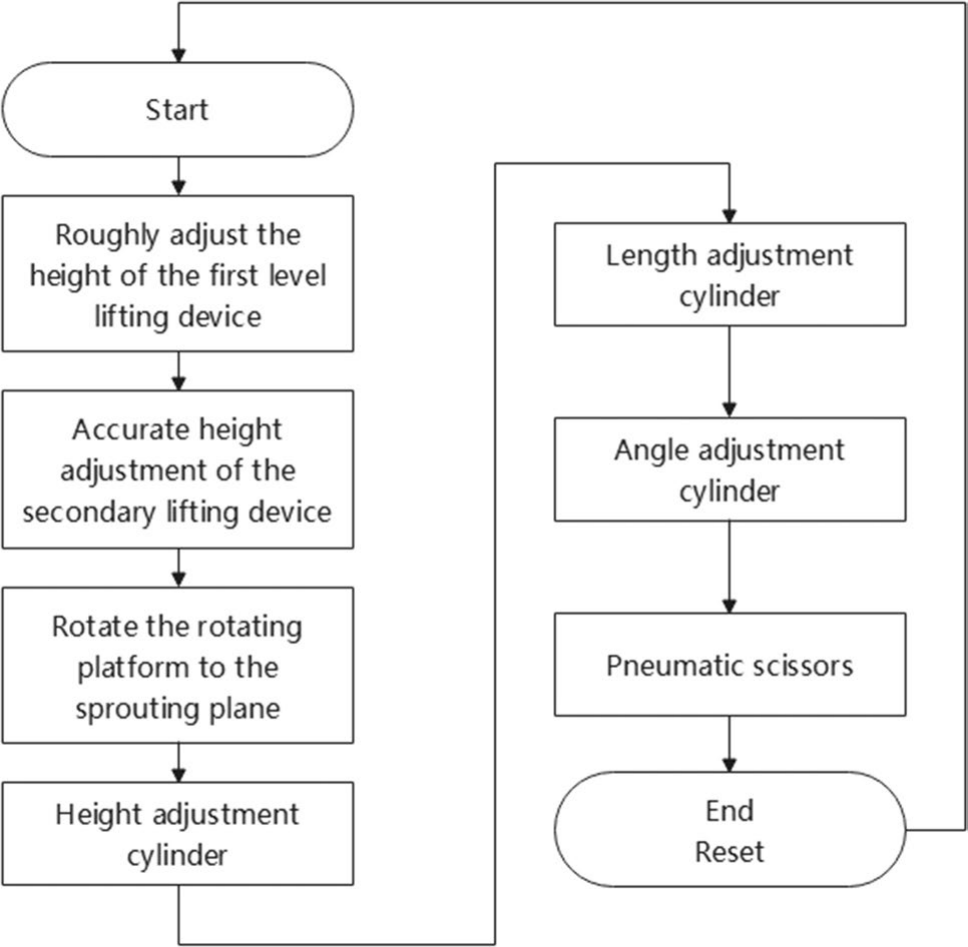
Control-flow diagram.

The PLC control process is illustrated in Fig. 15. The entire machine was controlled using the MCGS touchscreen jog control, which achieved precise positioning and cutting of the cutting position. The height, length, and angle adjustment cylinders determine their control sequences based on actual situations.

### Simulation and experimental verification

#### Kinematics simulation analysis of auxiliary resection device

The uncertainty of the direction of the pomegranate tree branches when they grow to form a disorganized and complex spatial structure leads to the complexity of the operating environment of this apparatus for cutting pomegranate sprouts. Therefore, when performing the operation, the target branch is prone to interference from the remaining branches. At the same time, the spatial movement of the robotic arm is restricted, making it difficult to contact the target branch to be removed. To eliminate the interference of other branches on the target branch and robotic arm, a pneumatic-assisted resection device was designed, which mainly consisted of a toggle cylinder, core toggle frame, toggle plate, and toggle seat. The toggle seat is fixed to the rotating disc of the device, and through the extension and contraction of the cylinder, the end of the core toggle holder is swung with the hinge connection with the toggle seat as the center, which finally achieves the swing of the toggle plate.

The most critical parts of the auxiliary cutting mechanism are the toggle cylinder and core toggle part. The motion in SolidWorks was used for the kinematic analysis of the toggle cylinder, and the simulation finite element analysis was used for the static analysis of the core toggle part. First, a DSNU-12-200-P-A cylinder was selected from the Festo series as the driving cylinder for the auxiliary device. In Motion, the cylinder was set as a linear motor driver, and the entire auxiliary device uses 45 # steel as the component material. Owing to the use of the inching control for the cylinder control, the speed of the driver was set to 30 mm/s, and the time to reach the maximum output was set to 6 s. The calculation results are illustrated separately, and the relationship between the speed, force on the linear motor (cylinder), and displacement with time is shown in Table 3(a–c).

**Table 3:**
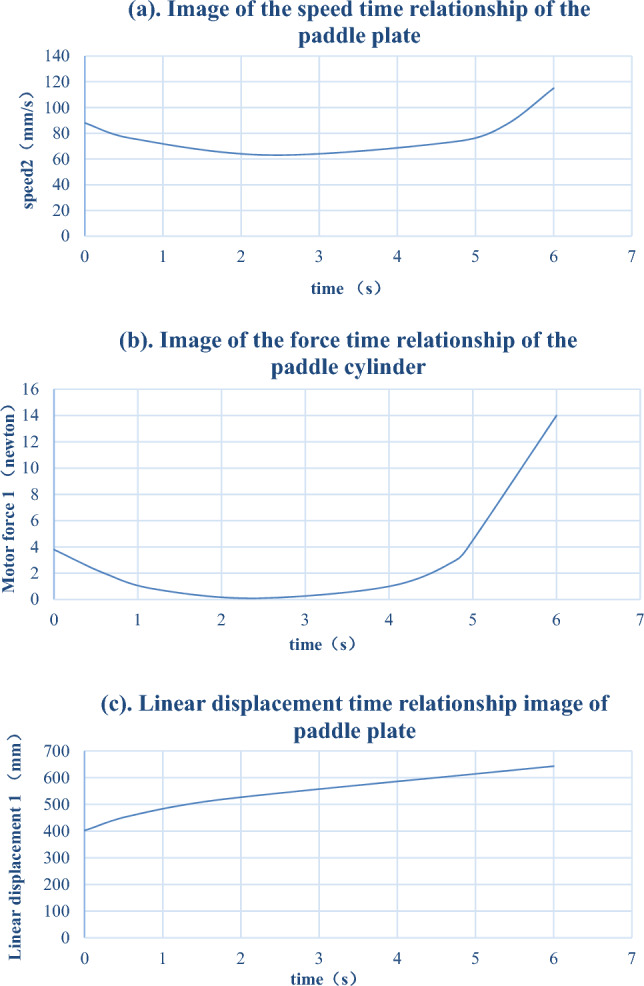
Speed time, force time, and linear displacement time relationship of the paddle plate.

From Table 3(a), it can be observed that the maximum speed of the paddle plate is 115 mm/s, which meets the requirement of moving and obstructing tree branches. From Table 3(c), it can be observed that the maximum force on the cylinder is 14 N, which occurs at the end of the cylinder movement. According to relevant manuals, the maximum force on the cylinder of the DSNU-12-200-P-A model when extending and retracting is 50.9 N and 67.9 N, respectively. Therefore, the selected model cylinder met its usage requirements and was reasonably designed.

#### Statics simulation analysis of auxiliary resection device

After completing the 3D modeling of the auxiliary cutting mechanism, mechanical analysis was performed on the relevant load-bearing parts. As mentioned earlier, the core component of the auxiliary cutting device was the core toggle frame. In order to ensure its stable force and no significant displacement deformation during operation, in this paper, the finite element simulation of the core toggle frame was carried out after the design of the part using ZWMeshworks2023 structural simulation software with Lanczos eigenvalue method solution matrix. Firstly, in ZWMeshworks2023, the model of the toggle leaf version in Solidworks is imported, and then a new linear static analysis task is created to set the toggle leaf version model material as AISI 1045 steel, and its physical properties are shown in Table 4.

**Table 4:** Physical properties of AISI 1045 steel materials.

Attribute	Numerical value	Unit
Elastic modulus	2.05 × 10^11^	N/m^2^
Poisson’s ratio	0.29	
Shear Modulus	8 × 10^10^	N/m^2^
Mass density	7850	kg/m^3^
Tensile strength	625,000,000	N/m^2^
Yield strength	530,000,000	N/m^2^
Thermal conductivity	49.8	W/(m K)
Specific heat	486	J/(kg K)

The network division of the dialleaf version is then carried out by ZWMeshworks2023, and the network division settings for the dialleaf version in this study are shown in Table 5, which are generated as shown in Fig. 17a.

**Table 5:** Parameters for dividing the network in the leaf-splitting version.

Property	Parameter
Minimum size	0.34715 mm
Maximum size	6.94298 mm
Tolerance	0.01
Maximum gradation	0.5
Chordal control type	Lsotopic
Element	Auto

**Figure 16:**
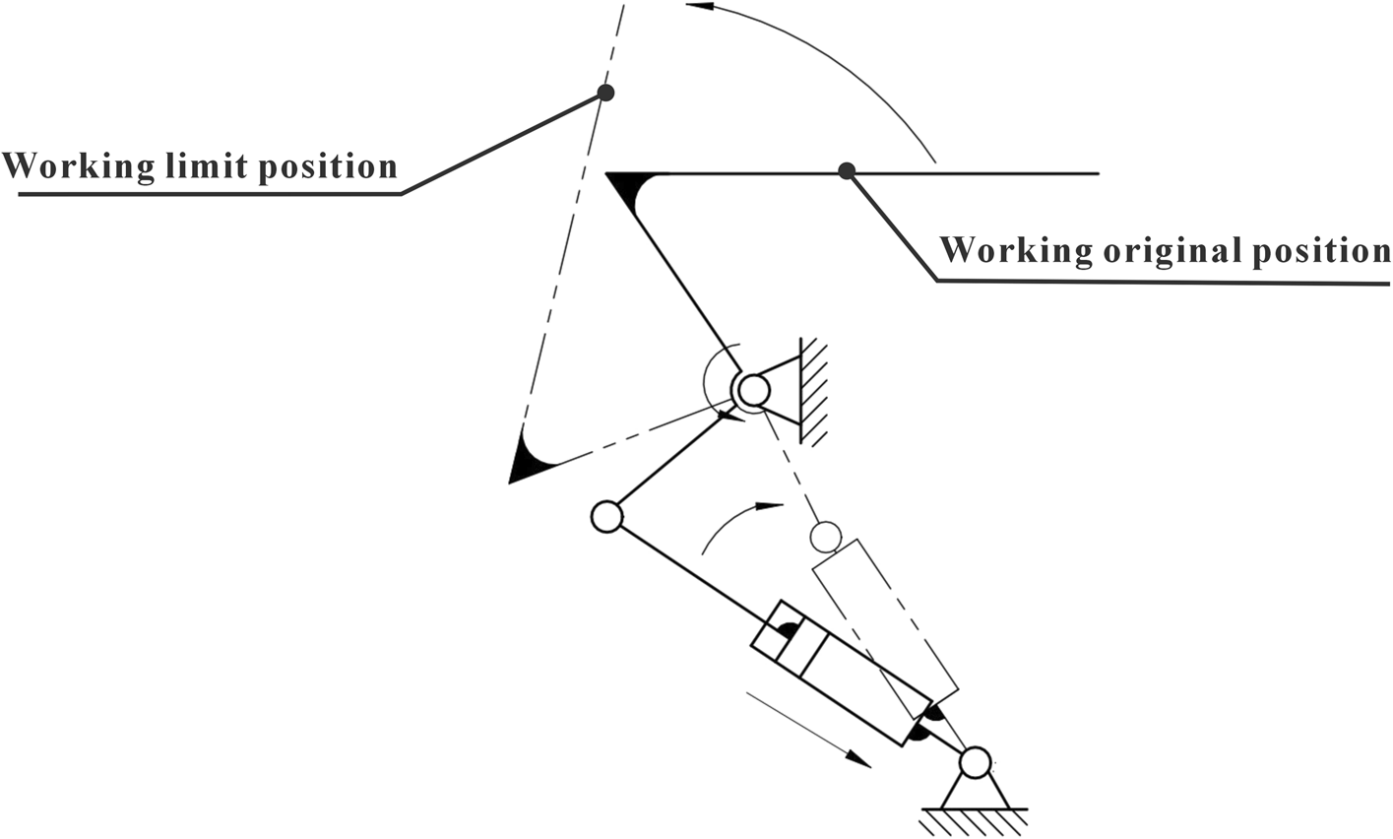
Motion sketch.

To better understand the force of this structural member in the device, as shown in Fig. 16. In this study, by constructing a motion sketch of this device, motion analysis of this device is carried out to more accurately set the mechanical load and constraint state of the core toggle frame.

Analyzing the entire motion process, it can be seen that in the initial state, the core toggle frame is subjected to the minimum force, and at this time, the cylinder has no thrust effect on the bottom end of the core toggle frame. When the movement begins, the gravity force of the toggle plate is converted to the pressure on the core toggle frame; at this time, the lower end of the push cylinder on the core toggle frame thrust gradually increases; when the movement to the highest height, the gravity force of the toggle plate is converted to the core toggle frame pressure; at this time, the cylinder extends to the longest height, and the thrust force reaches the maximum, solid at this time, the core toggle frame by the maximum force.

**Figure 17:**
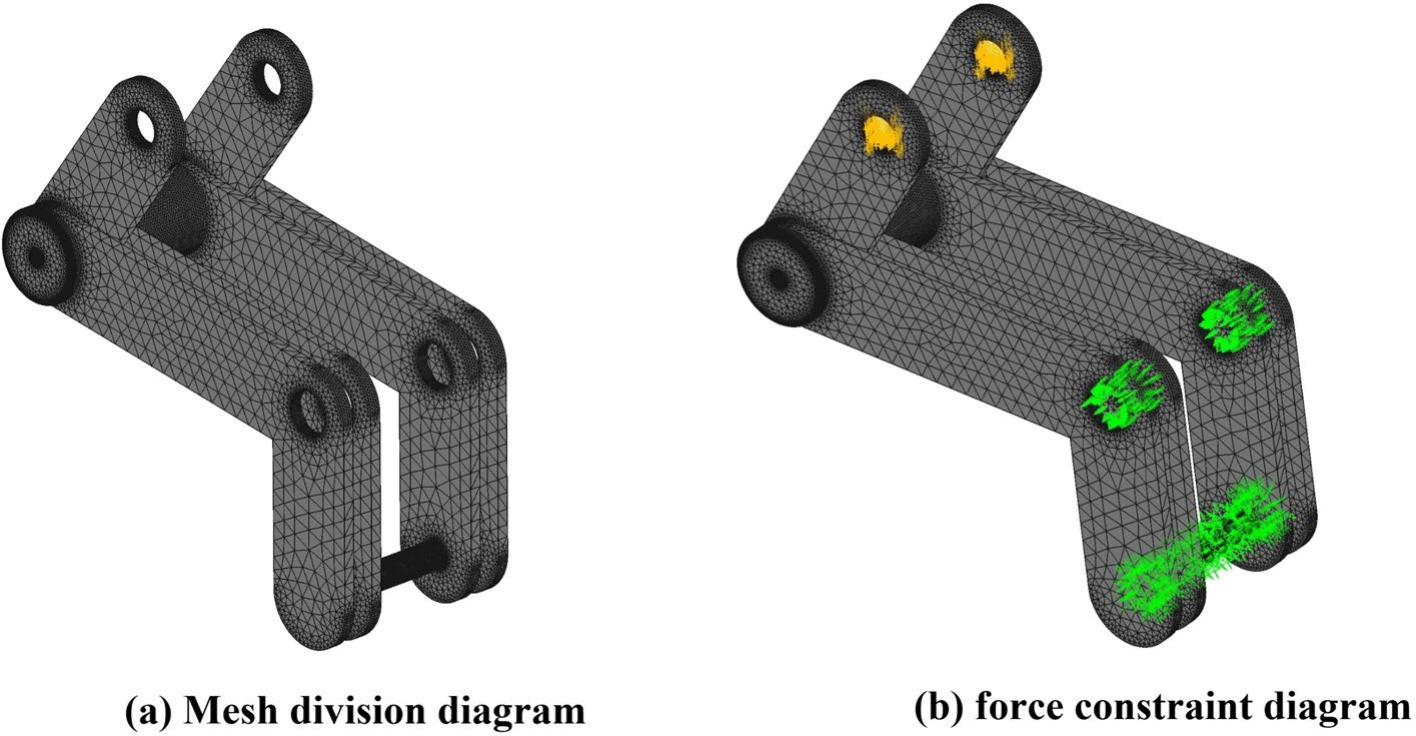
Mesh division diagram and force constraint diagram.

At the same time, in the working process, the core toggle frame middle hole position of the realization of the hinge is fixed and rotated around its position, as well as in the movement to promote the version of gravity on the maximum impact of this piece, ignoring the movement of the cylinder parts, only to analyze the linear displacement of the paddle plate to arrive at the limit value; the force received by the cylinder also reached a maximum value of 14N, the core toggle frame of the force, so this paper on the core toggle frame middle hole position set up hinged fixation in the connection between the cylinder and the core toggle frame to set up the fixed constraints, as shown in Fig. 17b.

**Figure 18:**
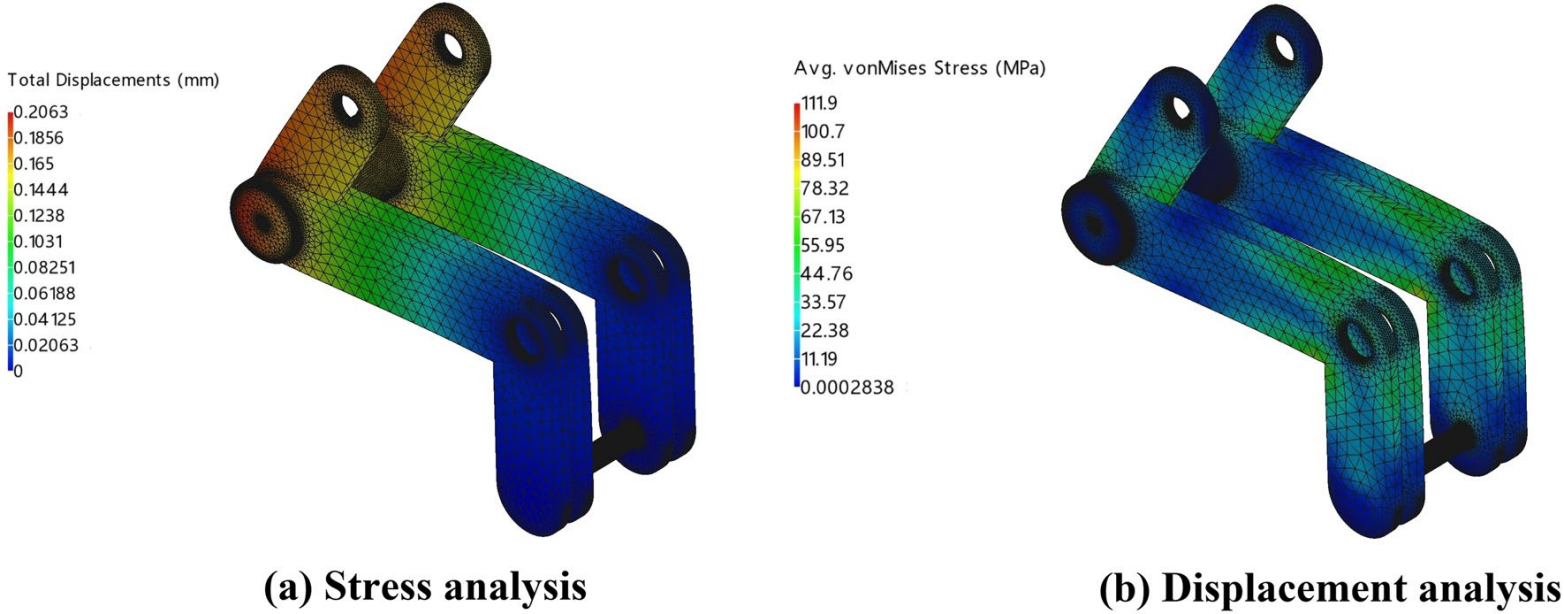
Stress analysis and displacement analysis.

Finally, the structural simulation results are calculated in ZWMeshWorks2023 for the processing as described above, and the results of the stress and displacement analyses are obtained, as shown in Fig. 18.

According to Fig. 18, the maximum stress on the core toggle frame was 111.9 MPa, and the maximum displacement of the core toggle frame during the stress was 0.2063 mm. According to the physical information of AISI 1045 steel in Table 4, the maximum stress on the core toggle frame is much less than the yield strength of AISI 1045 steel, and the strain is within the allowable range; therefore, the design of the solid-core toggle frame meets the usage requirements. requirements.

## Results

### Experimental materials, conditions, and equipment

**Figure 19:**
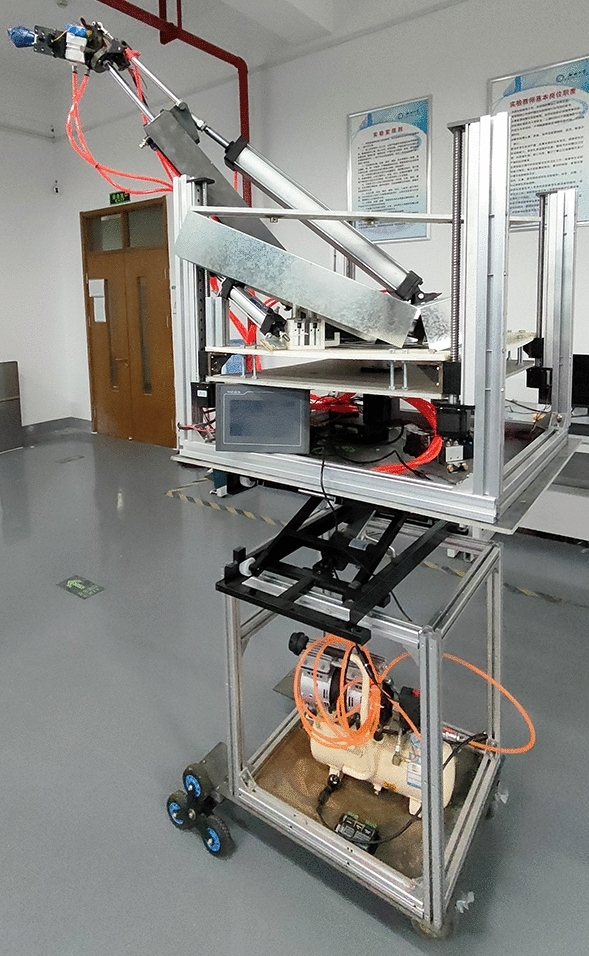
Physical prototype image of pomegranate sprout cutting machine.

To complete the final experiment of the machine and test whether the multi degree of freedom end actuator can work normally, a pruning test was conducted in the engineering training building of Liaocheng University in April 2023 for trees with similar heights to pomegranate trees. The height of the branches and leaves of the experimental trees was 2–3 m. The power was selected from the German Komax 220v small oil-free silent air compressor with a power of 1490w and a maximum pressure of 0.8 Mpa. The power supply was a 220–2500 W outdoor mobile power supply, which supplied power to the air pump and electric components in the machine. The selected prototype was the pomegranate sprout cutting device studied in this study, as shown in Fig. 19, and the measuring tool for measuring the diameter of sprouting branches was a tape measure.

### Evaluating indicator

The pruning rate and damage rate were selected as evaluation indicators to intuitively demonstrate the cutting effect and quality of the pruning machine. Each indicator is the average of multiple experiments and is defined as

11 $$P_{1} = \frac{Z_{1}}{Z}$$

In the formula, P_1_ represents the pruning rate of the entire tree's sprouts, %. Z_1_ is the actual number of sprouts cut by the machine. Z is the total number of sprouts that must be cut within the entire tree.

12 $$P_{2} = \frac{Z_{2}}{{Z_{3}}}$$

where P_2_ is the damage rate of non-sprouting branches of the entire tree,%. Z_2_ is the total number of damaged non sprouting branches. Z_3_ is the total number of non sprouting branches of the entire tree.

### Orthogonal experiment

To optimize the parameters and determine the interactivity between experimental factors, the range of experimental factors, such as sprouting diameter A, air pump working pressure B, and cylinder operating speed C, were determined through calculation and analysis. A three-factor, three-level quadratic regression orthogonal experiment was conducted with shear rate P_1_ and damage rate P_2_ as the response values. The experimental factor codes are listed in Table 5.

Using Design Expert software for data analysis and processing, the results of the three-factor and three-level experiments designed based on the Box–Behnken experimental principle are presented in Table 6.

**Table 6:** Encoding of experimental factors.

Code	Factor		
	Sprouting diameterA/(mm)	Air pump working pressureB/(Mpa)	Cylinder running speedC/(mm/s)
− 1	2	0.3	3.5
0	6	0.45	6
1	10	0.6	8.5

Using Design Expert software, multiple linear regression and quadratic fitting were performed on pruning rate P1 and damage rate P2 of pomegranate sprouts. The quadratic regression equations for P1 and P2 were obtained, as shown in Table 7.

13 $$\begin{aligned} & P1 = 67.32 - 4.8 \cdot A + 1.03 \cdot B - 4.35 \cdot C - 1.03 \cdot AB + 1.03 \cdot AC + 0.925 \cdot BC + 2.08 \cdot A^{2} \\ & \quad - 0.6725 \cdot B^{2} + 0.2775 \cdot C^{2} \\ \end{aligned}$$

14 $$\begin{aligned} & P2 = 19.86 - 0.775 \cdot A + 0.4 \cdot B - 5.23 \cdot C - 0.125 \cdot AB + 1.43 \cdot AC + 0.275 \cdot BC + 0.8425 \cdot A^{2} \\ & \quad - 0.4925 \cdot B^{2} + 3.46 \cdot C^{2} \\ \end{aligned}$$

**Table 7:** Orthogonal experimental scheme and results.

Number	Influence factor			Response factors	
	Sprouting diameterA/(mm)	Air pump working pressureB/(Mpa)	Cylinder running speedC/(mm/s)	Shear rateZ_1_/(%)	Damage rateZ_2_/(%)
1	10	0.6	6	63.1	18.1
2	6	0.45	6	67.1	19.6
3	6	0.45	6	67.6	19.9
4	6	0.3	8.5	63.7	26.5
5	6	0.45	6	67.4	20.3
6	2	0.45	3.5	79.3	16.4
7	10	0.45	3.5	72.1	17.2
8	6	0.6	8.5	64.8	28.7
9	2	0.45	8.5	65.2	30.6
10	10	0.45	8.5	62.1	25.7
11	6	0.3	3.5	70.9	17.5
12	2	0.3	6	72.3	19.2
13	2	0.6	6	79.2	18.9
14	6	0.6	3.5	68.3	18.6
15	6	0.45	6	67.3	19.7
16	6	0.45	6	67.2	19.8
17	10	0.3	6	60.3	17.9

Using Design Expert software for the regression equation analysis of variance simulation, Tables 6 and 7 were obtained. From Table 8, it can be seen that the quadratic regression model of the pruning rate of the pomegranate sprout cutting machine (*P* < 0.05) indicated that the regression model was significant. Analysis of variance showed that the sprout diameter (*P* < 0.05) had a significant impact on the shear rate. The cylinder running speed (*P* < 0.05) indicates that the cylinder running speed has a significant impact on the shear rate. Analysis of variance shows that the significance of each factor on the shear rate is in descending order: sprout diameter and cylinder running speed. The working pressure of the air pump had no significant impact.

**Table 8:** Analysis of variance of regression equation of shear clean rate.

Source	Sum of squares	*Df*	Mean square	F-value	*P*-value	
Model	375.98	9	41.78	3.75	0.0475	Significant
Sprouting diameter	184.32	1	184.32	16.57	0.0047	
Air pump working pressure	8.41	1	8.41	0.7555	0.4136	
Cylinder running speed	151.38	1	151.38	13.61	0.0078	
AB	4.20	1	4.20	0.3777	0.5583	
AC	4.20	1	4.20	0.3777	0.5583	
BC	3.42	1	3.42	0.3076	0.5964	
A^2^	18.17	1	18.17	1.63	0.2420	
B^2^	1.90	1	1.90	0.1712	0.6915	
C^2^	0.3242	1	0.3242	0.0291	0.8693	

From Table 9, it can be seen that the quadratic regression model for the damage rate of the pomegranate sprout cutting machine (*P* < 0.0001) indicated that the regression model was extremely significant. Analysis of variance showed that sprout diameter (*P* < 0.05) had a significant impact on the damage rate. A cylinder operating speed of *P* < 0.0001 indicated that the cylinder operating speed had a significant impact on the damage rate. Analysis of variance showed that the significance of each factor on the shear rate was in the following descending order: air cylinder operating speed and sprout diameter. The working pressure of the air pump had no significant impact. The AC interaction term has a significant impact, proving that there is an interaction between the sprout diameter and cylinder operating speed on the damage rate.

**Table 9:** Analysis of variance of injury rate regression equation.

Source	Sum of squares	*Df*	Mean square	F-value	*P*-value	
Model	285.66	9	31.74	57.61	< 0.0001	Significant
Sprouting diameter	4.80	1	4.80	8.72	0.0213	
Air pump working pressure	1.28	1	1.28	2.32	0.1713	
Cylinder running speed	218.40	1	218.40	396.38	< 0.0001	
AB	0.0625	1	0.0625	0.1134	0.7461	
AC	8.12	1	8.12	14.74	0.0064	
BC	0.3025	1	0.3025	0.5490	0.4828	
3 A^2^	2.99	1	2.99	5.42	0.0527	
B^2^	1.02	1	1.02	1.85	0.2156	
C^2^	50.33	1	50.33	91.35	< 0.0001	

### Response surface analysis

**Figure 20:**
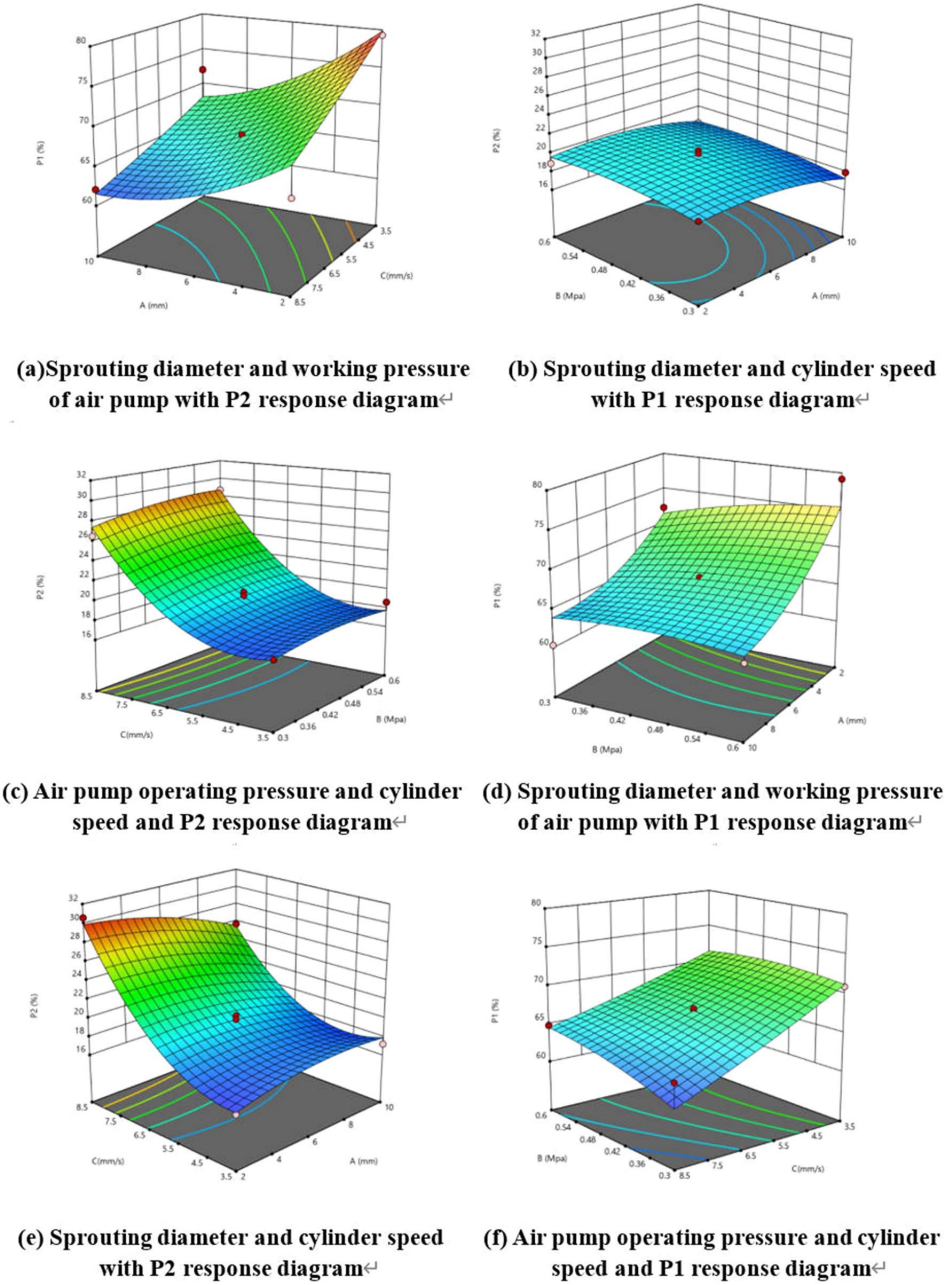
Response surface analysis.

Using Design Expert software for solution optimization analysis, the optimal solution was obtained, which was achieved when the average diameter of the sprouts was 2.684 mm, working pressure of the air pump was 0.442 Mpa, and speed of cylinder movement was 5.068 mm/s. At this time, the shear rate was 74.622% and the damage rate was 18.004%. Using the values at this point as standard values and as the variation values under the interaction in response surface analysis, the response surfaces under the influence of each interaction can be obtained, as shown in Fig. 20a–f.

From the response surface analysis in the above figure, it can be seen that the variation pattern of the factors affecting the response surface is basically consistent with the results of the model and regression equation variance analysis; in other words, the interaction term between sprout diameter A, C cylinder motion speed, and AC has a significant impact on the removal rate and damage rate. The working pressure of air pump B has no significant impact on either. Finally, the two optimal indicators of the machine, namely the shear rate of 74.622% and damage rate of 18.004%, were also determined.

## Discussion

This paper presents a systematic description of the overall structure and workflow of a machine that consists of a camera that determines the position of the pomegranate sprouting tiller and transmits it to a display screen, a primary and secondary lifting platform, a multi-degree-of-freedom end-effector, and an auxiliary leaf-shifting device. Simulations and finite element analyses were performed to determine the structural soundness of this study. The most critical part of the auxiliary cutting mechanism is the toggle cylinder and the core toggle part of the simulation analysis. The maximum stress of the core toggle frame is 5.127 × 10^7^ N/m^2^, and the maximum displacement of the core toggle frame during the stress process is 1.281 × 10^−2^ mm. The maximum stress of the core frame was much less than the permissible stress of the AISI 1045 steel material, and the strain displacement was within the permissible range. Finite element analysis was used to determine the average diameter of the buds at 2.684 mm, the working pressure of the air pump was 0.442 Mpa, and the cylinder movement speed was 5.068 mm/s. At this time, to determine the machine's two best indicators, namely 74.622% of the shear rate and 18.004% of the damage rate. Experimental data show that the machine can greatly reduce the labor force, liberate the hands of fruit farmers, and has broad application prospects.

## Conclusion

### Author's statement

A PLC-based pomegranate bud cutting device was proposed and designed to address the background of the low efficiency of pomegranate bud high branch cutting, high labor occupation by repetitive manual labor, and high risk factors. The machine structure was designed independently by the author of this study and the feasibility of the machine was experimentally measured. The content of this paper was independently completed by the author of this paper.

The technological innovation and advantages of this device are:

1. This device designs a multi degree of freedom pneumatic end effector, which cooperates with three cylinders: length adjustment cylinder, angle adjustment cylinder, and height adjustment cylinder, to achieve multi angle and all-round cutting of pomegranate sprouts under complex growth conditions, achieving full mechanization of the sprout cutting process and greatly reducing the labor required.
2. This device is designed with a multilevel height adjustment mechanism that controls the expansion and contraction of the cylinder to raise and lower the pneumatic platform, thus completing the first-level rough lifting of the device. The second-level lifting device used four stepper motor screw modules to control the stepper motor for precise height adjustment. The mutual cooperation of the first- and second-level lifting devices can achieve complete removal of pomegranate sprouts within different height ranges.
3. This device designs a sprout cutting auxiliary leaf-shifting device with dual-cylinder coordination, which enables the device to complete the sprout cutting of branches that need to be trimmed without damaging the main stem of the pomegranate tree.
4. The entire control system of the device was controlled by the MCGS. Through the MCGS, it can control the stepper motor, cylinder, and other executing components of the entire device, and display its motion process and status. The entire execution mechanism is controlled by only one screen and related buttons, thereby enhancing the flexibility and practicality of the entire machine operation.

## Data Availability

The datasets used and analyzed during the current study are available from the corresponding author upon reasonable request.
